# Supplementary material for: Long-Term Adverse Events Following Early Breast Cancer Treatment with a Focus on the BRCA-Mutated Population
Source: Cancers (Basel). 2025 Jul 30;17(15):2506. doi: 10.3390/cancers17152506 (PMC12345669; doi:10.3390/cancers17152506)
Supplement: Supplementary file 1 [file cancers-17-02506-s001.zip › cancers-3722697-supplementary.pdf]

Review

Long-Term Adverse Events Following Early Breast Cancer Treatment with a Focus on the *BRCA*-Mutated Population

Supplementary Material

Supplementary Table S1. Summary of key clinical studies reporting on long-term adverse events n patients with BC.

Author(s)	Year	Study design	Sample Size	Long-term adverse effects considered
Pierce *et al.* (1)	2000	Retrospective cohort	284	Skin lesions
Boneterre *et al.* (2)	2004	Prospective	150	Cardiovascular events
Shanley *et al.* (3)	2006	Retrospective case-control	110	Cardiovascular and osteomuscular alterations
Ganz *et al.* (4)	2008	Retrospective	180	Cardiovascular events
Rabaglio *et al.* (5)	2009	Phase III, randomized, double-blind clinical trial	4,895	Osteomuscular alterations
Cuzick *et al.* (6)	2010	Randomized controlled trial	6,186	Osteomuscular alterations
Carmody *et al.* (7)	2011	Randomized controlled trial	110	Hormonal alterations
Tanabe *et al.* (8)	2013	Retrospective	117	Neurological events
Valentini *et al.* (9)	2013	Retrospective	795	Sexual disorders
Park *et al.* (10)	2014	Retrospective	213	Skin lesions
Aerts *et al.* (11)	2014	Prospective	149	Sexual disorders
Eckhoff *et al.* (12)	2015	Retrospective	NR	Neurological events
Bao *et al.* (13)	2016	Cross-sectional	296	Neurological toxicity
Barac *et al.* (14)	2016	Prospective	81	Cardiovascular events
Drooger *et al.* (15)	2016	Retrospective	701	Gastrointestinal toxicities
Abrahams *et al.* (16)	2016	Meta-analysis	12.327	Fatigue
Forbes *et al.* (17)	2016	Double-blind, randomized controlled trial	2,938	Osteomuscular, endocrine, and neurological alterations
Pearson *et al.* (18)	2017	Retrospective cohort	102	Cardiovascular events
Sajjad *et al.* (19)	2017	Exploratory	79	Cardiovascular events
Litton *et al.* (20)	2018	Phase 3, open-label, randomized trial	431	Fatigue, neurological, and gastrointestinal toxicities
Lange *et al.* (21)	2020	Prospective	264	Neurological toxicity
Wang *et al.* (22)	2020	Meta-analysis	282,203	Psychological alterations
Johnston *et al.* (23)	2020	Open-label, randomized, phase III trial	5,637	Fatigue and gastrointestinal toxicity
Khosrow-Khavar *et al.* (24)	2020	Population-based cohort study	NR	Cardiovascular events
Tutt *et al.* (25)	2021	Randomized controlled trial	1836	Fatigue
Di Meglio *et al.* (26)	2021	Prospective, multicenter, clinical study of stage I-III	6.619	Fatigue
Partridge *et al.* (27)	2021	Single-arm, prospective, international cohort study	518	Fertility and reproductive outcomes
Poorvu *et al.* (28)	2021	Prospective	1,026	Fertility and reproductive outcomes

Puckett *et al.* (29)	2021	Single-arm, feasibility (prospective) study	3201	Cardiovascular toxicity
Sund *et al.* (30)	2021	Retrospective	15,815	Cardiovascular events
Geyer *et al.* (31)	2022	Phase III, double-blind, randomized trial	1,836	Neurological and gastrointestinal toxicities
Carmen *et al.* (32)	2022	Prospective	268	Hormonal alterations and fertility
Rugo *et al.* (33)	2022	Phase III, open-label, randomized, study	5,591	Gastrointestinal toxicity
Vliek *et al.* (34)	2023	Randomized controlled trial	NR	Fatigue and gastrointestinal toxicity
Gao *et al.* (35)	2023	Prospective	292	Fatigue, psychological alterations, and gastrointestinal toxicity
Hurvitz *et al.* (36)	2023	Randomized controlled trial phase II	221	Fatigue and gastrointestinal alterations
Digkas *et al.* (37)	2024	population- and registry-based study	21,268	Endocrine toxicity
Huang *et al.* (38)	2024	Double-blind, randomized controlled Trial phase III	329	Gastrointestinal toxicity
Roy *et al.* (39)	2024	Retrospective	2,215	Cardiovascular events
Connors *et al.* (40)	2025	Retrospective	240	Gastrointestinal, skin, neurological, and endocrine alterations

NR: not reported. From the complete list of studies cited in the manuscript, the most pertinent clinical studies detailing long-term adverse events in patients with breast cancer were selected and summarized in the Table.

Supplementary References:

1. L. J. Pierce *et al.*, Effect of radiotherapy after breast-conserving treatment in women with breast cancer and germline BRCA1/2 mutations. *J Clin Oncol* **18**, 3360-3369 (2000).

2. J. Bonneterre *et al.*, Long-term cardiac follow-up in relapse-free patients after six courses of fluorouracil, epirubicin, and cyclophosphamide, with either 50 or 100 mg of epirubicin, as adjuvant therapy for node-positive breast cancer: French adjuvant study group. *J Clin Oncol* **22**, 3070-3079 (2004).

3. S. Shanley *et al.*, Late toxicity is not increased in BRCA1/BRCA2 mutation carriers undergoing breast radiotherapy in the United Kingdom. *Clin Cancer Res* **12**, 7025-7032 (2006).

4. P. A. Ganz *et al.*, Late cardiac effects of adjuvant chemotherapy in breast cancer survivors treated on Southwest Oncology Group protocol s8897. *J Clin Oncol* **26**, 1223-1230 (2008).

5. M. Rabaglio *et al.*, Bone fractures among postmenopausal patients with endocrine-responsive early breast cancer treated with 5 years of letrozole or tamoxifen in the BIG 1-98 trial. *Ann Oncol* **20**, 1489-1498 (2009).

6. J. Cuzick *et al.*, Effect of anastrozole and tamoxifen as adjuvant treatment for early-stage breast cancer: 10-year analysis of the ATAC trial. *Lancet Oncol* **11**, 1135-1141 (2010).

7. J. F. Carmody *et al.*, Mindfulness training for coping with hot flashes: results of a randomized trial. *Menopause* **18**, 611-620 (2011).

8. Y. Tanabe *et al.*, Paclitaxel-induced peripheral neuropathy in patients receiving adjuvant chemotherapy for breast cancer. *Int J Clin Oncol* **18**, 132-138 (2013).

9. A. Valentini *et al.*, Chemotherapy-induced amenorrhea in patients with breast cancer with a BRCA1 or BRCA2 mutation. *J Clin Oncol* **31**, 3914-3919 (2013).

10. H. Park *et al.*, Acute skin toxicity in Korean breast cancer patients carrying BRCA mutations. *Int J Radiat Biol* **90**, 90-94 (2014).

11. L. Aerts, M. R. Christiaens, P. Enzlin, P. Neven, F. Amant, Sexual functioning in women after mastectomy versus breast conserving therapy for early-stage breast cancer: a prospective controlled study. *Breast* **23**, 629-636 (2014).

12. L. Eckhoff, A. Knoop, M. B. Jensen, M. Ewertz, Persistence of docetaxel-induced neuropathy and impact on quality of life among breast cancer survivors. *Eur J Cancer* **51**, 292-300 (2015).

13. T. Bao *et al.*, Long-term chemotherapy-induced peripheral neuropathy among breast cancer survivors: prevalence, risk factors, and fall risk. *Breast Cancer Res Treat* **159**, 327-333 (2016).

14. A. Barac *et al.*, Cardiac function in BRCA1/2 mutation carriers with history of breast cancer treated with anthracyclines. *Breast Cancer Res Treat* **155**, 285-293 (2016).

15. J. C. Drooger *et al.*, Toxicity of (neo)adjuvant chemotherapy for BRCA1- and BRCA2-associated breast cancer. *Breast Cancer Res Treat* **156**, 557-566 (2016).

16. H. J. G. Abrahams *et al.*, Risk factors, prevalence, and course of severe fatigue after breast cancer treatment: a meta-analysis involving 12 327 breast cancer survivors. *Ann Oncol* **27**, 965-974 (2016).

17. J. F. Forbes *et al.*, Anastrozole versus tamoxifen for the prevention of locoregional and contralateral breast cancer in postmenopausal women with locally excised ductal carcinoma in situ (IBIS-II DCIS): a double-blind, randomised controlled trial. *Lancet* **387**, 866-873 (2016).

18. E. J. Pearson, A. Nair, Y. Daoud, J. L. Blum, The incidence of cardiomyopathy in BRCA1 and BRCA2 mutation carriers after anthracycline-based adjuvant chemotherapy. *Breast Cancer Res Treat* **162**, 59-67 (2017).

19. M. Sajjad *et al.*, An exploratory study to determine whether BRCA1 and BRCA2 mutation carriers have higher risk of cardiac toxicity. *Genes (Basel)* **8**, 59 (2017).

20. J. K. Litton *et al.*, Talazoparib in patients with advanced breast cancer and a germline BRCA mutation. *N Engl J Med* **379**, 753-763 (2018).

21. M. Lange *et al.*, Cognitive impairment in patients with breast cancer before surgery: results from a CANTO cohort subgroup. *Cancer Epidemiol Biomarkers Prev* **29**, 1759-1766 (2020).

22. X. Wang *et al.*, Prognostic value of depression and anxiety on breast cancer recurrence and mortality: a systematic review and meta-analysis of 282,203 patients. *Mol Psychiatry* **25**, 3186-3197 (2020).

23. S. R. D. Johnston *et al.*, Abemaciclib combined with endocrine therapy for the adjuvant treatment of HR+, HER2-, node-positive, high-risk, early breast cancer (monarchE). *J Clin Oncol* **38**, 3987-3998 (2020).

24. F. Khosrow-Khavar, K. B. Filion, N. Bouganim, S. Suissa, L. Azoulay, Aromatase inhibitors and the risk of cardiovascular outcomes in women with breast cancer: a population-based cohort study. *Circulation* **141**, 549-559 (2020).

25. A. N. J. Tutt *et al.*, Adjuvant olaparib for patients with BRCA1- or BRCA2-mutated breast cancer. *N Engl J Med* **384**, 2394-2405 (2021).

26. H. J. Di Meglio A, Martin e, Pistilli B, Menvielle G, Dumas A, Charles C, Everhard S, Martin AL, Countant C, Tarpin C, Vanlemmens L, Levy C, Rigal O, Delaloge S, Ganz PA, Partridge AH, Andre F, Michiels S, Vaz Duarte Luis IM, Assessing the risk of severe post-treatment (tx) cancer-related fatigue (CRF) among breast cancer survivors (BCS) in the CANcer TOxicity (CANTO) cohort. *J Clin Oncol* **39**, 12022 (2021).

27. A. H. Partridge *et al.*, Who are the women who enrolled in the POSITIVE trial: a global study to support young hormone receptor positive breast cancer survivors desiring pregnancy. *Breast* **59**, 327-338 (2021).

28. P. D. Poorvu *et al.*, Pregnancy after breast cancer: results from a prospective cohort of young women with breast cancer. *Cancer* **127**, 1021-1028 (2021).

29. L. L. Puckett *et al.*, Cardiotoxicity screening of long-term, breast cancer survivors - The CAROLE (Cardiac-Related Oncologic Late Effects) study. *Cancer Med* **10**, 5051-5061 (2021).

30. M. Sund *et al.*, Aromatase inhibitors use and risk for cardiovascular disease in breast cancer patients: A population-based cohort study. *Breast* **59**, 157-164 (2021).

31. C. E. Geyer, Jr. *et al.*, Overall survival in the OlympiA phase III trial of adjuvant olaparib in patients with germline pathogenic variants in BRCA1/2 and high-risk, early breast cancer. *Ann Oncol* **33**, 1250-1268 (2022).

32. A. Carmen *et al.*, Does the toxicity of endocrine therapy persist into long-term survivorship?: Patient-reported outcome results from a follow-up study beyond a 10-year-survival. *Breast Cancer Res Treat* **198**, 475-485 (2022).

33. H. S. Rugo *et al.*, Adjuvant abemaciclib combined with endocrine therapy for high-risk early breast cancer: safety and patient-reported outcomes from the monarchE study. *Ann Oncol* **33**, 616-627 (2022).

34. S. Vliek *et al.*, High-dose alkylating chemotherapy in BRCA-altered triple-negative breast cancer: the randomized phase III NeoTN trial. *NPJ Breast Cancer* **9**, 75 (2023).

35. Y. Gao *et al.*, Longitudinal changes of health-related quality of life over 10 years in breast cancer patients treated with radiotherapy following breast-conserving surgery. *Qual Life Res* **32**, 2639-2652 (2023).

36. S. A. Hurvitz *et al.*, Neoadjuvant palbociclib plus either giredestrant or anastrozole in oestrogen receptor-positive, HER2-negative, early breast cancer (coopERA Breast Cancer): an open-label, randomised, controlled, phase 2 study. *Lancet Oncol* **24**, 1029-1041 (2023).

37. E. Digkas *et al.*, Incidence and risk factors of hypothyroidism after treatment for early breast cancer: a population-based cohort study. *Breast Cancer Res Treat* **204**, 79-87 (2024).

38. L. Huang *et al.*, Neoadjuvant-adjuvant pertuzumab in HER2-positive early breast cancer: final analysis of the randomized phase III PEONY trial. *Nat Commun* **15**, 2153 (2024).

39. S. Roy *et al.*, Major cardiovascular adverse events in older adults with early-stage triple-negative breast cancer treated with adjuvant taxane + anthracycline versus taxane-based chemotherapy regimens: A SEER-medicare study. *Eur J Cancer* **196**, 113426 (2024).

40. C. Connors *et al.*, Real-world outcomes with the KEYNOTE-522 regimen in early-stage triple-negative breast cancer. *Ann Surg Oncol* **32**, 912-921 (2025).
